# Rheumatic Carditis in Childhood

**Published:** 1907-09

**Authors:** Carey Coombs

**Affiliations:** Demonstrator of Pathology, University College, Bristol; Assistant Physician, Bristol General Hospital.


### The Bristol Medico-Chirurgical Journal.

*"Scire est nescire, nisi id me

Scire alius sciret."*

SEPTEMBER, 1907.

### RHEUMATIC CARDITIS IN CHILDHOOD. √

BY

CAREY COOMBS, M.D. Lond.,

*Demonstrator of Pathology, University College, Bristol;*
*Assistant Physician, Bristol General Hospital.*

THESE remarks are prompted by a survey of four recent autopsies and sixty-five cases seen during the past few years. For my pathological notes I am indebted to Dr. Michell Clarke, Dr. Cecil Williams, and Dr. T. E. Holmes. Of the clinical cases twenty-four are my own out-patients, and the remainder in-patients at St. Mary's Hospital and at the Children's Hospital, Bristol, the notes of which I am able to use by the kindness of those physicians under whom the children were admitted. The in-patients show a larger percentage of severe types of carditis than the out-patients, as one might expect. Of seventy-five children seen in Bristol with various rheumatic disorders, forty-five, or 60 per cent., had rheumatic carditis. Forty of the cases are girls, twenty-five boys; the boys are relatively more liable to the severer forms of rheumatic heart disease. The age at which the first evidence of rheumatic infection was seen varied from $3\frac{1}{2}$ to 14, and averaged 8. In only six, at the most, did the cardiac symptoms precede other rheumatic phenomena.

The pathology of rheumatic carditis presents four salient points. *First*, it is a carditis; for example, in my four autopsies the endocardium and myocardium were inflamed in each instance, and the pericardium three times.[1] *Second*, the rheumatic infection is blood-borne, and attacks the heart just as it would any other organ, by way of its own special nutritional blood supply, in this instance the coronary arteries. That this is so is suggested by the simultaneous invasion of all three layers of the cardiac wall and by the position of the lesions, periarterial in the muscular and primarily subendothelial in the serous layers. Further, acute arteritis and other infective lesions may be met with in the coronary arteries in rheumatic carditis. In my four cases there was subendothelial fatty change at the root of the aorta, in the area fed from the coronary arteries. *Third*, the gravity of rheumatic carditis in the earliest or "childhood" stage lies mainly in the damage done to the muscle. This is on a priori grounds to be expected; the heart's main functions are muscular, and the serous layers clothing the myocardium are merely accessories to the easy and effective performance of those functions. Both clinically and after death the great feature of acute rheumatic carditis is ventricular dilatation, as has been so ably shown by Dr. Lees and Poynton.[2] In later years the permanent serous lesions become important; by that time the myocardium shows little trace either in its histology or in its functional capabilities of the damage inflicted upon it at the time of the invasion. *Fourth*, rheumatic carditis, like all rheumatic lesions, is remarkably apt to recur—or should it be said to recrudesce? Perhaps the infective agent lies quiet in the cardiac tissues awaiting opportunity to reassert itself; perhaps it is stored elsewhere in the body, and issues thence at intervals to infect the heart again. It is worthy of remark that in all my four autopsies (and in others I have seen)

1 For larger statistics see Poynton, *Med.-Chir. Tr.*, 1899, lxxxii. 355. This paper is referred to later in this article.

2 *Med. Chir. Tr.*, 1898, lxxxi. 419.

the interbronchial lymph-glands were much enlarged. Be this as it may, certain it is that the recurrences lead to the establishment of permanent fibrosis, eventually more important in the serous than in the muscular layers; in the early acute stages, however, it is the myocarditis that kills.

This way of regarding rheumatic heart disease, that is, as a carditis principally important in childhood by reason of the muscular lesions, is confirmed by clinical experience. Of my sixty-five cases, no less than thirty-eight presented certain physical signs about to be described; and of the remaining twenty-seven, ten were of the same kind, but the signs were incompletely developed. They were like enough to those in the larger group, however, to make one think them due to the same pathological process occurring in a less severe degree.

The typical physical signs are as follows. On inspection, the impulse is seen over an area extending from the left sternal border, or even from the right of the sternum, to an inch or more outside the left mammary line, and from the third left space above to the fifth or sixth left space below. It is usually possible to see that the impulse is wavelike or peristaltic in character; the wave begins at the sternal ends of the third and fourth left spaces, and travels downwards and outwards to disappear outside the nipple in the fifth or sixth left space. As it reaches this latter extreme an apparent sinking-in is noticed in those spaces where the wave was first seen. This gives an impression of systolic retraction, yet it is in reality not a retraction but merely a rebound, the chest-wall falling back into the position which it occupied before it was thrust forward by the contracting heart. The point of maximum impulse is at or external to the left mammary line, usually in the fifth space; here it is so firm and well-sustained in the average quiescent case as to prove the presence of ventricular hypertrophy. At the inner end of the third left space a sharp diastolic shock is felt, presumably due to an abrupt forcible closure of the pulmonary valves under the influence of hypertension in the lesser circulation. Sometimes the peristalsis I have described is so vibratile as to give the impression of a presystolic thrill; this, however, is never so rough, powerful and definite as the thrill of mitral stenosis. I have felt a systolic thrill at and external to the apex. The deep cardiac dulness is transversely increased both to right and to left ; it may even extend from the right mammary line to the left mid-axillary line.

The auscultatory signs at the apex show a certain progress. The first departure from the normal is a lengthening and blurring of the first sound ; then a definite systolic bruit develops after the first sound, which may finally replace it. This is transmitted into the axilla and, of course, indicates mitral leakage. Next the apical second sound becomes doubled, and then, but often not till some time after, a murmur is added to the second half of this second sound. This, hard to distinguish at first, lengthens and strengthens till at last it runs into the beginning of the next cycle, becoming, in fact, a presystolic murmur. This is not as rough and loud as that of mitral obstruction, and it is not due to valvular disease.

At the base the only noteworthy feature is a tremendous accentuation of the pulmonic second sound, which is often doubled also ; the explanation of this accentuation is, of course, the usual one—raised tension in the pulmonary artery.

Now these signs—outward displacement of the apex-beat, widened area of impulse, abnormally ready perception of the peristaltic nature of systole, increase of the transverse cardiac dulness on both sides, the murmur of mitral incompetence, and the signs of raised pulmonary tension—are all due to a dilatation of both ventricles. That there is hypertrophy is suggested by the firm character of the impulse at its maximum point ; but the principal feature is dilatation. The ventricles have stretched because their walls are atonic, and the atony is due to the action of the rheumatic virus upon the myocardium.

From time to time the dilatation, which has been partially recovered from in the interval, is acutely exaggerated by a fresh infection of the heart muscle ; not infrequently this is so damaging to the myocardial cells as to lead to a fatal asystole. *Post-mortem*, the heart is enlarged transversely and globular, owing to ventricular dilatation ; the auriculo-ventricular orifices are stretched, and the ventricular walls are thickened. The muscle may be pale, but naked-eye changes are usually insignificant. There may or may not be evidences of pericardial or endocardial infection; more often than not such lesions are present, but of no great gravity. Sections of the myocardium show perivascular areas of leucocytosis with proliferative inflammation of the stroma, leading eventually to a permanent fibrosis; and in the parenchyma loss of striation and fatty change.[1]

Three of the cases I examined *post-mortem* had during life presented those signs which have been already described; in two of them there was in addition a pericardial rub, and in these two there was an early fibrinous pericarditis. In all three the valves were inflamed—the mitral in three, the aorta in two, and tricuspid in one; the lesions were of the usual type, circumscribed nodules capped with a little fibrin. In none of the three, however, were these lesions sufficient to account for death, or even for cardiac embarrassment; but in all three there was considerable ventricular dilatation, associated with some thickening of the muscle, and with the microscopical appearances already mentioned as occurring in the myocardium.

In the fourth case there were similar myocardial and valvular changes, but in addition there were lesions of the pericardium, which during life had notably altered the physical signs from those already described. The modifications which may thus arise from special types of inflammatory change in the serous layers remain to be outlined.

In both endocardium and pericardium the reaction to rheumatic infection may for convenience' sake be divided into three stages: the acute, the recurrent, and the cicatricial. It is with the first two that childhood is mainly concerned. In the pericardium (to consider the first) there is a more or less acute reaction to the first invasion, which occurs, of course, by way of the coronary circulation; as this subsides the fibrin which has been thrown down is partly reabsorbed and partly used as the basis for new fibrous tissue. Recurrences (or recrudescences) take place repeatedly, and the pericardial sac is gradually obliterated; but

1 See papers by Poynton (*Lancet*, 1900, i. 1352), Fisher (*Lancet*, 1902, i. 1594), West, Herringham, Carpenter and others.

it is only when there are also pericardio-mediastinal adhesions that really grave and specific effects are produced, and in such cases cardiac failure is much more likely to occur than in the absence of this complication. Clinically, the acute is not the commonest phase of rheumatic pericarditis in childhood: only one true example occurred in my sixty-five cases. It is characterised by rapid increase in the area of cardiac dulness, which is due to ventricular dilatation, and not to effusion of fluid (which, as Poynton's figures show, is rare, if not unknown, in rheumatic carditis), and by a friction sound over the whole præcordium. It appears to terminate in resolution, but in reality it leads to the first instalment of those obliterating adhesions already spoken of.

The recurrent stage is commonest clinically; it was exemplified by six of my cases, five of whom were boys. Slight pyrexia, with dyspnœa and perhaps cardiac pain, leads to an examination of the heart, which is found dilated; friction sounds are heard, often for a day or two only, over a limited fraction of the præcordium, and the whole attack soon subsides. Probably its real nature is often overlooked, especially as the rub may be pleuro-pericardial and respiratory in rhythm.

There was no good example of the cicatricial stage among my sixty-five children; adhesions strong and dense enough to produce systolic recession take so long in building that childhood is gone before signs appear. My fourth autopsy was, however, upon a case of this type, a boy of 13, admitted to the Bristol General Hospital under Dr. Michell Clarke. The heart was encased in a dense mat of adhesions, fully half an inch thick over the ventricles. Myocarditis was proved by microscopy, and the valves were beaded. The adhesions bound the pericardial layers together and the heart as a whole to the surrounding tissues. This boy had during life shown unmistakable systolic recession.

Rheumatic infection of the endocardium is usually limited by the local reactive processes to the deeper parts of the valves, where it arrives by way of the coronary circulation; circumscribed nodules are formed, and permanent cicatrisation generally, if not invariably, follows. Sometimes, however, the resistance is lowered either locally by preformed fibrosis, or generally by some malnutritive state, and the organisms find their way to the surface of the valve, forming an infective ulcer, " pointing," in fact, into the cardiac cavity. How is the first type, the commoner circumscribed form of endocarditis, to be linked with the extreme cicatrisation seen in ordinary mitral stenosis ? Is there constant irritation by a latent infection, or are there repeated reinfections ? This cannot as yet be answered ; all that can be definitely said is that advanced cicatrisation is not common before the age of 16.

Clinically, the circumscribed type of rheumatic endocarditis is perceptible in its acute stage only when the aortic valves are swollen, and by no means always then. The only evidence is a diastolic bruit, usually very indistinct, for the cicatricial stage must be reached before the ordinary signs of aortic insufficiency are noted. In at least one of my cases an aortic regurgitant murmur appeared and disappeared again in so short a time as to leave an impression of subsiding inflammation which had temporarily crippled the valve. A similar affection of the mitral valve produces no special signs, as the valve is already incompetent by reason of myocardial weakness. The systolic murmur is myocardial, and not endocardial in its origin.

The malignant type of rheumatic endocarditis was not represented among my cases. Figures bearing on its frequency will be found in the writings of Osler[1] and Glynn.[2]

The recurrent and cicatricial stages of rheumatic endocarditis make themselves apparent only by the disablement of the valves which they produce. For all practical purposes we may consider aortic incompetence and mitral obstruction as the two perversions of the valvular functions resulting from such disablement ; and neither of these conditions is common, in well-developed form, in childhood. A presystolic murmur and thrill do not necessarily mean mitral stenosis in rheumatic children. *Post-mortem*, the valves are usually found to show evidence of early fibrosis, but the condition is quite unworthy to be classed as " stenosis." It is not surprising, therefore, that in none of my cases was an unmistakable stenosis present. No doubt many of [illegible]

[1] Goulstonian Lectures, 1885. [2] Lumleian Le[illegible]

show it in later years; but it had not had time to develop at 16.

Aortic incompetence was present in thirteen out of my sixty-five cases, but this must not be taken to indicate a preponderance of aortic over mitral lesions as the effect of rheumatism. It simply means that the aortic valves are easily and quickly rendered incompetent, while it needs a good deal of fibrosis to obstruct the mitral orifice. As a matter of fact, rheumatism attacks the mitral valves nearly three times as often as the aortic (Poynton), a figure supported by some observations of my own. Of 155 cases of valvular disease examined by me at St. Mary's Hospital, there were 99 purely mitral cases, 34 purely aortic; of the former 82 per cent. gave a history of acute rheumatism, of the latter 35 per cent. only. The remainder were cases of combined aortic and mitral disease, of whom 77 per cent. gave a similar history.

There is no space here for a discussion of the prognosis or of the lines of treatment. The object of the paper is to show that for the production of those signs which are met with in the great majority of cases of rheumatic carditis in childhood it is quite unnecessary to blame the valves or the pericardium; the myocardial changes which are always present are enough to account for everything. There are, however, modifications of the fundamental clinical picture due to special types of inflammatory change in the serous layers. Of these the commonest are aortic incompetence and recurrent pericarditis, which, I may add, not infrequently coincide in the same patient.

